# A New Hope for Woman with Vasomotor Symptoms: Neurokinin B Antagonists

**DOI:** 10.3390/jcm14051438

**Published:** 2025-02-21

**Authors:** Blazej Meczekalski, Anna Kostrzak, Christian Unogu, Stefania Bochynska, Marzena Maciejewska-Jeske, Gregory Bala, Anna Szeliga

**Affiliations:** 1 Department of Gynecological Endocrinology, Poznan University of Medical Sciences, 60-535 Poznan, Poland; 2 UCD School of Medicine, University College Dublin, D04 V1W8 Dublin, Ireland

**Keywords:** NKB, neurokinin B, elinzanetant, fezolinetant, menopause, vasomotor symptoms

## Abstract

KNDy (kisspeptine, neurokinin B, dynorphin) neurons, located in the hypothalamus, play a crucial role in the development of vasomotor symptoms (VSM) in menopausal women. Estrogen withdrawal during menopause leads to the hyperactivation of kisspeptin and neurokinin B (NKB) secretion, contributing to the onset of these symptoms. The identification of NKB/neurokinin B receptor (NK3R) signaling as a key mechanism in menopausal hot flashes has driven the development of NK3R antagonists. These antagonists restore the disrupted balance in KNDy neuron activity caused by estrogen deficiency, thereby reducing the frequency and severity of VMS. In 2023, the FDA approved fezolinetant, the first selective NK3R antagonist, for the treatment of moderate to severe VMS associated with menopause. Additionally, elinzanetant, a dual neurokinin-1 and neurokinin-3 receptor antagonist, has demonstrated promising results. The approval application for elinzanetant was supported by positive findings from the OASIS 1, 2, and 3 Phase III clinical studies. The dual antagonism of NK-1 and NK-3 receptors enhances its efficacy by alleviating menopause-related sleep disturbances and modulating peripheral vasodilatation. In this regard, elinzanetant represents a promising non-hormonal treatment that targets the underlying causes of VMS through NK-1 and NK-3 receptor pathways. The development of neurokinin B antagonist for VMS treatment exemplifies the impact of advanced pharmacological research on gynecological endocrinology.

## 1. Introduction

Menopause, defined as the permanent cessation of menstruation due to a decline in ovarian hormonal function, represents a natural stage in a woman’s life and is characterized by significant physiological and psychological changes. This transition, which includes both the perimenopausal and postmenopausal phases, is often accompanied by a range of symptoms that can profoundly impact quality of life. Hormonal fluctuations, particularly the decline in estrogen levels, are the primary drivers of symptoms, such as hot flashes, sleep disturbances, mood swings, and cognitive difficulties, including memory and concentration issues. Moreover, menopause is associated with an increased risk of chronic conditions, including osteoporosis, cardiovascular diseases, and metabolic disorders [1].

In recent years, significant progress has been made in understanding the mechanisms underlying menopause and its associated symptoms. Central to this growing body of knowledge is the role of KNDy neurons—comprising Kisspeptin, Neurokinin B, Dynorphin—located in the hypothalamus. These neurons play a pivotal role in thermoregulation and gonadotropin hormone release. Dysregulation of KNDy neurons, often linked to estrogen deficiency, has been identified as a key mechanism driving hot flashes, one of the most distressing symptoms of menopause. Research on KNDy neurons has facilitated the development of targeted therapies, offering promising prospects for improving quality of life during this life stage [2].

This paper aims to provide a contemporary perspective on menopause, integrating current knowledge of its biological mechanisms with the latest therapeutic advancements. Notably, NKB antagonists represent a significant breakthrough in VMS treatment, particularly for patient groups with contraindications to hormonal therapy. Special emphasis is placed on the role of KNDy neurons in the pathophysiology of menopausal symptoms and their potential for pharmacological intervention. Additionally, this review discusses other modern therapeutic strategies, including personalized medicine and emerging innovative technologies. Given the aging population and the increasing number of women at the age of menopause, understanding and effectively managing this stage of life is both a medical necessity and a matter of considerable social and economic importance [3].

Further research and innovation in this field hold the potential not only to improve the quality of life for women experiencing menopause but also to enhance our broader understanding of aging processes and their impact on women’s health.

### Methods

This narrative review involved a comprehensive search of several major databases, including PubMed, ScienceDirect, Excerpta Medica Database, UpToDate, and the Cochrane Library. The search strategy employed a combination of MeSH terms and free-text keywords, including “NKB”, “neurokinin B”, “neurokinin B antagonist”, “KNDy”, “fezolinetant”, “elinzanetant”, and “menopause”. Searches were conducted between 1 November 2024 and 30 November 2024. All identified publications in English up to November 2024 were critically appraised by the authors, with a specific focus on studies directly related to the primary topic.

Two authors independently reviewed the titles and abstracts, systematically classifying them based on strict inclusion criteria. Duplicates, conference proceedings, and editorial letters were excluded. Full texts of clinical studies, review articles, and meta-analyses were then thoroughly examined and evaluated. Additionally, reference lists of the included articles were manually screened to identify further relevant studies.

## 2. Overview of KNDy Neurons: Anatomy and Function

Kisspeptin is a neuropeptide encoded by the *KISS1* gene in humans [4]. The *KISS1* gene was first identified during studies investigating the varying metastatic potential of human melanoma cells [5].

Recently, a novel hypothesis has emerged, highlighting a group of neurons located in the arcuate nucleus (ARC) that co-express kisspeptin, neurokinin B (NKB), and dynorphin (Dyn), collectively referred to as KNDy neurons. These neurons play a critical role in the episodic release of gonadotropin-releasing hormone (GnRH) into the hypophysial portal vessels, thereby regulating the pulsatile secretion of luteinizing hormone (LH) [6].

There are three neurokinin receptor subtypes—NK1R, NK2R, and NK3R—encoded by the *Tacr1*, *Tacr2*, and *Tacr3* genes, respectively. Studies have shown that *Tacr3* is located on chromosome 8 [7]. Expression of NK3R has been primarily observed in the central nervous system, with *Tacr3* mRNA detected in the hippocampus, hypothalamus, pituitary gland, and spinal cord but not in peripheral tissues [8]. Additionally, NK3 is also present in the human ovary, uterus, kidney, and lung.

The first direct evidence linking kisspeptin to GnRH, the central regulator of reproduction, was described in a study on tilapia (*Oreochromis niloticus*), where expression of the *gpr54* gene was demonstrated in all three types of GnRH neurons in the species. In the human hypothalamus, kisspeptin neurons are primarily located in two regions: the anteroventral periventricular nucleus (AVPV) and the infundibular nucleus. Kisspeptin neurons in the AVPV act as a surge generator, while KNDy neurons in the infundibular nucleus function as a pulse generator. A proposed model delineates specific roles for the three KNDy peptides: kisspeptin serves as the output signal to GnRH neurons, NKB initiates each pulse within the KNDy network, and dynorphin acts on KNDy cells to terminate the pulse [9].

The preoptic area (PO) of the hypothalamus serves as a homeostatic control center in humans. QPLOT neurons—characterized by the expression of pyroglutamylated Arg-Phe-amide peptide (QRFP), prostaglandin EP3 receptor (*Ptger3*), leptin receptor (*LepR*), violet light sensing Opsin 5 (*Opn5*), and the neurokinin B receptor NK3R encoded by (*Tacr3*)—integrate various stimuli to regulate thermogenesis and metabolism. QPLOT neurons play an integral role in modulating body temperature and energy expenditure [10].

**Figure 1:**
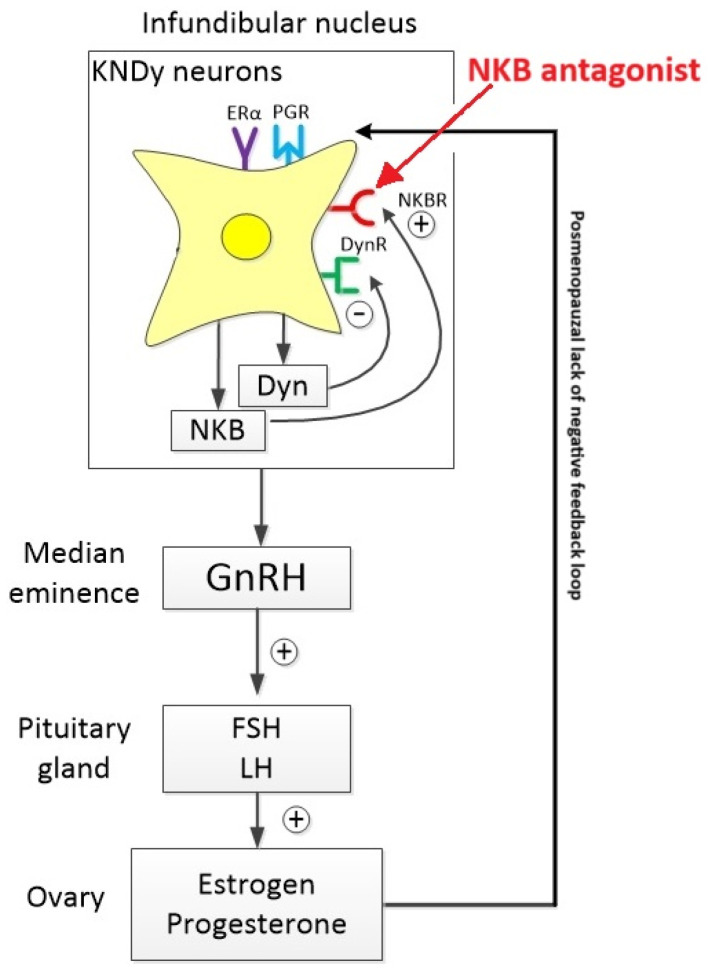
Anatomy of KNDy neurons. Neurokinin B (NKB) synthesized in KNDy neurons stimulates GnRH neurons, promoting GnRH production, which in turn stimulates the secretion of FSH and LH in response to low estradiol concentrations. NKB antagonists inhibit the hyperactivation of KNDy neurons in postmenopausal patients, restoring normal signaling in the hypothalamus and preventing dysregulated vasodilation. Abbreviations: NKB—Neurokinin B, Dyn—dynorphin, DynR—Dynorphin receptor, NKBR—Neurokinin B receptor, PG—progesterone receptor, ERα—Estradiol receptor α.

KNDy neurons have also been implicated in the generation of vasomotor symptoms, particularly hot flashes, in menopausal women. Estrogen withdrawal during menopause induces changes in gene expression within the hypothalamus, leading to the dysregulation of KNDy neurons and contributing to the manifestation of these symptoms (Figure 1).

Since the initial discovery of the relationship between NKB and reproductive processes, numerous studies have been conducted to explore its regulatory role within the hypothalamic–pituitary–ovary (HPO) axis and its underlying mechanisms [11]. NKB is now broadly recognized as a key upstream regulator of GnRH secretion and, consequently, a fundamental regulator of the HPO axis.

## 3. History of Past Research on KNDy Analogs in Menopause Treatment

KNDy neurons, located in the hypothalamus, are critical regulators of reproductive function. These neurons co-express three essential neuropeptides: kisspeptin, neurokinin B, and dynorphin. Together, they orchestrate the pulsatile release of GnRH, a process essential for regulating the reproductive cycle [1,2]. NKB activates KNDy neurons, prompting the release of kisspeptin, which stimulates GnRH secretion, while dynorphin acts as an inhibitory signal to terminate the GnRH pulse [12]. This foundational understanding has been refined through extensive cellular studies, demonstrating that KNDy neurons are both necessary and sufficient for generating the pulsatile GnRH secretion crucial for reproductive function [13].

Emerging research highlights the intricate network of interactions, including the role of the excitatory neurotransmitter glutamate, that synchronizes KNDy neuron activity within the arcuate nucleus—a key hypothalamic region involved in hormonal regulation [14]. These findings have paved the way for the development of innovative, non-hormonal treatments for menopausal symptoms.

Beyond their role in reproductive function, KNDy neurons also regulate body temperature, energy balance, and body weight [15,16]. During menopause, the decline in estrogen levels disrupts the equilibrium of KNDy neurons, resulting in heightened activity and increased NKB signaling [17,18]. It is believed that this overactivation contributes significantly to the development of VMS, such as hot flashes and night sweats [19]. The altered signaling dynamics within the KNDy network lead to inappropriate activation of heat dissipation pathways, disrupting thermoregulation. Early animal studies have provided key insights into this connection, demonstrating that activation of NKB receptors (NK3R) in the median preoptic nucleus (MnPO), a key thermoregulatory center, induces heat dissipation responses resembling menopausal hot flashes [15,20].

These findings are further supported by human studies that identified genetic variations in the NK3R gene associated with an increased risk of VMS, further establishing the connection between NKB/NK3R signaling and menopausal hot flashes [21,22]. Recently, high-resolution 3D structures of NK3R bound to various agonists have revealed unique binding properties and activation mechanisms, offering critical insights for the development of targeted therapies, such as the synthetic agonist senktide [23].

The identification of NKB/NK3R signaling as a central mechanism in menopausal hot flashes has led to the development of NK3R antagonists as a promising non-hormonal treatment option [24,25]. These antagonists work by restoring the disrupted balance in KNDy neuron activity caused by estrogen deficiency, thereby restoring the frequency and severity of VMS. By selectively targeting NK3R, these agents mitigate overactive NKB signaling and re-establish thermoregulatory stability [26]. Fezolinetant, a leading NK3R antagonist, has shown remarkable efficacy in both preclinical and clinical studies [27]. In animal models, it successfully prevented the rise in core body temperature associated with hot flashes [28,29,30]. Clinical trials in menopausal women have further demonstrated that fezolinetant significantly reduces the frequency and severity of hot flashes, leading to its recent approval as a treatment for moderate to severe VMS associated with menopause [31,32].

While NK3R antagonists remain the primary focus, there is growing interest in the therapeutic potential of KNDy analogs. Although kisspeptin antagonists are less extensively studied in the context of menopause, they have the potential to offer additional treatment avenues. Kisspeptin agonists have already shown promise in treating certain forms of infertility by stimulating GnRH release [33]. Given that kisspeptin regulates GnRH release through its action on LH and FSH secretion, blocking kisspeptin activity could help stabilize hormonal fluctuations associated with menopause [34]. Additionally, research into dynorphin analogs and kappa opioid receptor agonists has demonstrated potential for modulating KNDy neuron activity and alleviating hot flashes [35]. Preclinical studies and trials in postmenopausal women have shown that kappa opioid agonists stimulate the dynorphin pathway, leading to improvements in body temperature regulation, LH levels, and hot flash frequency [36,37].

KNDy neurons play a central role in both reproductive function and thermoregulation. Disruption in their signaling, particularly elevated NKB activity, contributes to menopausal hot flashes. NK3R antagonists, such as fezolinetant, represent a promising non-hormonal treatment for vasomotor symptoms. Further research into KNDy analogs holds significant potential for developing additional targeted therapies to improve the quality of life for menopausal women.

## 4. Fezolinetant—A New Hope for Women with Vasomotor Symptoms (VMS)

In 2023, the U.S. Food and Drug Administration (FDA) approved fezolinetant, the first neurokinin 3 (NK-3) receptor antagonist indicated for the treatment of moderate to severe vasomotor symptoms (VMS) associated with menopause [32]. Clinical trials have demonstrated that fezolinetant significantly reduces both the frequency and severity of VMS compared to placebo [38].

Many women have contraindications to hormone therapy or chose to decline it due to potential associated risks. Absolute contraindications for hormone therapy include unexplained vaginal bleeding, liver disease, a history of estrogen-sensitive cancer, and a history of or high risk for thromboembolic disease, coronary heart disease, stroke, or myocardial infarction. Moreover, hormone therapy is not recommended for women over 60 years of age or those who are ≥10 years post-menopause due to an unfavorable risk–benefit profile. For such patients, fezolinetant offers a promising, non-hormonal alternative [39].

Studies on diverse populations representative of the target demographic for fezolinetant therapy support its clinical use. In a 12-week study followed by 40-week blinded extension, fezolinetant demonstrated sustained efficacy and safety as a non-hormonal treatment for VMS associated with menopause [38,40]. Fezolinetant treatment is associated with improvement in overall menopause-specific quality of life and work productivity, measured by the work productivity and activity impairment questionnaire specific to VMS. A high proportion receiving fezolinetant felt VMS was ‘much better’ based on patient global impression of change in VMS responder analysis [41].

Fezolinetant is administered as a 45 mg oral tablet taken once daily, with or without food, as food does not affect its absorption [39]. Following administration, fezolinetant reaches the peak serum concentration within 1.5 h, and steady-state levels are achieved after two doses in healthy women. No dosage adjustments are required for patients with mild-to-moderate renal impairment; however, fezolinetant is contraindicated in those with severe renal impairment [39].

The most common adverse effects associated with fezolinetant include headache, gastrointestinal disturbances (e.g., abdominal pain and diarrhea), insomnia, back pain, and elevated hepatic transaminase levels [39,42]. In preregistration clinical trials, serum ALT or AST elevations exceeding three times the upper limit of normal were observed in 2.3% of patients receiving fezolinetant, compared to 0.9% in the placebo group. These elevations were typically asymptomatic, transient, and resolved after discontinuation of the drug. Higher doses of fezolinetant were associated with greater aminotransferase elevations, although associated bilirubin elevation was uncommon, and no cases of clinically significant liver injury with jaundice were reported in preregistration studies, even with high doses [42].

Despite this favorable safety profile, a post-registration case of mixed cholestatic hepatitis with symptoms and jaundice was reported. The adverse event occurred 54 days after initiating fezolinetant and resolved following drug discontinuation [42,43].

Given the observed impact on liver function, baseline bloodwork to assess hepatic function is recommended before starting fezolinetant treatment. Follow-up bloodwork should be conducted at 3, 6, and 9 months after initiation, as adverse liver events are most likely to occur within the first few months of therapy [38,42]. Additional blood tests should be performed if symptoms suggest liver injury.

As of May 2024, IQVIA Total Patient Tracker data indicates that approximately 28,700 patients have been prescribed Veozah (fezolinetant) through U.S. outpatient retail pharmacies [42].

## 5. Elinzanetant

The development of modern non-steroidal therapies for menopausal vasomotor symptoms (VMS) remains a critical area of investigation. A significant milestone was reached on 9 October 2024 when the U.S. Food and Drug Administration (FDA) [44] granted market access to elinzanetant for the treatment of moderate to severe hot flushes associated with menopause [45]. At the time of writing, elinzanetant is not yet commercially available; however, it remains a promising investigational non-hormonal treatment for menopausal VMS. It acts as a dual antagonist of the neurokinin-1 (NK-1) and neurokinin-3 (NK-3) receptors. The *SWITCH-1* trial—a multicenter, multinational, double-blind, phase IIb adaptive dose-ranging study—demonstrated that elinzanetant 120 mg significantly reduced the frequency and severity of VMS compared to placebo at 4 weeks (difference in least square means [SE]: −3.93 [1.02]; *p* < 0.001) and 12 weeks (−2.95 [1.15]; *p* = 0.01). The study also reported clinically meaningful improvements in sleep quality and overall quality of life [46]. Elinzanetant is expected to become commercially available in 2025 [47,48].

Elinzanetant is the first dual NK-1 and NK-3 receptor antagonist to receive FDA approval. This approval was supported by positive results from the OASIS-1, OASIS-2, and OASIS-3 Phase III clinical studies [48]. OASIS-1 and OASIS-2 specifically evaluated the efficacy and safety of elinzanetant when administered orally once daily in postmenopausal women experiencing moderate to severe VMS [49]. The studies enrolled 396 and 400 postmenopausal women, respectively, aged 40–65 years, across 184 sites in 15 countries. Participants in the treatment arm received 120 mg of elinzanetant once daily for 26 weeks, while the control group received a matching placebo for 12 weeks, followed by 120 mg of elinzanetant for an additional 14 weeks. The results demonstrated a significant reduction in VMS [49]:

- At week 4, VMS frequency decreased by 55.9% and 57.9% in OASIS-1 and OASIS-2, respectively, compared to placebo reductions of 31.4% and 35.7%.
- At week 12, VMS frequency decreased further, to 65.2% and 67.0%, compared to placebo reductions of 42.2% and 45.9% [49].

In addition to alleviating VMS, elinzanetant significantly improved sleep disorders and quality of life by week 12. Importantly, the treatment was well-tolerated, with no major adverse effects reported [48].

The OASIS-3 study further evaluated the long-term efficacy and safety of elinzanetant over 52 weeks in 628 postmenopausal women aged 40–65 years. The results confirmed the findings of OASIS-1 and OASIS-2. Notably, no cases of endometrial hyperplasia, endometrial cancer, or hepatotoxicity were reported, reinforcing the favorable safety profile of the drug. The OASIS-4 study focused on assessing the efficacy and safety of elinzanetant in women experiencing moderate to severe VMS as a result of endocrine therapy for breast cancer treatment or prevention.

Another notable advancement in the treatment of menopausal VMS is fezolinetant. The U.S. FDA approved fezolinetant on 13 May 2023 for the treatment of moderate to severe VMS associated with menopause [39]. Fezolinetant is notable as the first non-hormonal NK-3 receptor (NK3R) antagonist approved for this indication. However, as of 12 September 2024, the FDA issued a warning regarding the rare occurrence of serious liver injury associated with felizanetant use [43].

In comparison, elinzanetant offers a potential advantage due to its dual mechanism of action as both an NK-3 receptor and NK-1 receptor antagonist. The additional antagonism of NK-1 receptor may enhance its efficacy by modulating menopause-related sleep disturbances [46] and peripheral vasodilatation. In this regard, elinzanetant represents a promising non-hormonal option that targets the underlying cause of VMS through NK-1 and NK-3 receptor pathways.

The findings of Simon et al. (2023) [47] further support this hypothesis. Their study demonstrated that elinzanetant, at both 120 mg and 160 mg, significantly improved VMS, sleep disturbances, and quality of life in women experiencing menopausal symptoms [49].

The introduction of felizanetant and elinzanetant as non-hormonal treatments for menopause represents a significant advancement in the field. Clinical trial data indicate that elinzanetant is not associated with significant adverse effects; the most commonly reported side effects include headache and somnolence [49]. Both fezolinetant and elinzanetant are associated with beneficial outcomes in menopausal women with vasomotor symptoms. Elinzanetant provided a larger effect size in vasomotor symptom frequency and severity reduction and greatly improved sleep quality compared with fezolinetant [50].

Current and future research into non-hormonal treatments, innovative therapies, and methods to delay onset of menopause offer a positive outlook for women managing menopausal symptoms. These advancements hold great promise for improving quality of life and expanding treatment options for women worldwide.

## 6. Potential Use of Medications Targeting KNDy Neurons in Other Diseases

Vasomotor symptoms (VMS) are associated with significant morbidity and adverse health consequences in postmenopausal women. While hormone therapy remains the gold standard for treating VMS, it is not suitable for all patients due to contraindications or personal preference [51]. For these individuals, non-hormonal therapies, such as neurokinin receptor antagonists, offer a promising alternative by targeting KNDy neurons. Given their mechanism of action, neurokinin receptor antagonists may also have therapeutic applications in other reproductive disorders.

Neurokinin receptor antagonists could play a role in the treatment of polycystic ovary syndrome (PCOS), a prevalent endocrine disorder characterized by hyperandrogenism and an elevated LH-to-FSH ratio [51]. Research suggests that increased hypothalamic neurokinin B (NKB) levels contribute to hyperactive LH pulse secretion in PCOS [52]. By modulating KNDy neurons, neurokinin receptor antagonists may suppress this hyperactivity, subsequently reducing hyperandrogenism and improving hormonal balance in affected individuals [51].

These antagonists also show promise in the management of uterine fibroids and endometriosis, conditions that are currently treated with GnRH modulators to suppress the hypothalamic–pituitary–gonadal axis. The neurokinin-3 receptor (NK3R) represents an alternative therapeutic target due to its crucial role in modulating this axis. In preclinical studies, systemic administration of an NK3R antagonist, ESN364, prolonged the LH interpulse interval in ovariectomized ewes and significantly reduced plasma LH and FSH concentrations in castrated nonhuman primates (*Macaca fascicularis*). Daily oral administration of ESN364 throughout the menstrual cycle in *M. fascicularis* lowered plasma estradiol levels in a dose-dependent manner. Although estradiol levels remained above menopausal thresholds, suppression during the follicular phase effectively inhibited ovulation, as evidenced by the absence of an LH surge and luteal phase progesterone rise. Additionally, no significant alteration in FSH levels were observed outside of the LH surge. Importantly, these effects were reversible upon cessation of treatment, further supporting the role of neurokinin B-NK3R signaling in controlling pulsatile GnRH secretion [51,53].

While these findings highlight the potential of neurokinin receptor antagonists as therapeutic agents for reproductive disorders, further research is essential to comprehensively evaluate their efficacy and safety. Future studies must assess the clinical benefits of these treatments against any potential adverse effects to determine their suitability for broader clinical applications.

## 7. Discussion

By 2030, the global population of women experiencing menopause is projected to reach 1.2 billion, with an additional 47 million women entering menopause annually [54]. This projection underscores the urgent need to address menopause-related issues from an epidemiological and public health perspective.

Menopause is a natural physiological process characterized by the depletion of ovarian function and a subsequent decline in serum estradiol levels. It is associated with a range of symptoms, among which VMS are among the most distressing for patients. These symptoms primarily result from profound hypoestrogenism. For decades, the mainstay of VMS treatment has been hormonal menopausal therapy (HMT), including estrogen or estrogen-progestin therapy, which replenishes the hormones that decline during menopause. However, despite its widespread use, the precise mechanisms underlying VMS remained unclear until recent advancements in understanding its pathophysiology.

The discovery of kisspeptin marked a groundbreaking milestone in endocrinology, revolutionizing the understanding of reproductive function regulation, diagnostics, and treatment [55]. This progress was further amplified by the identification of KNDy neurons, which play a pivotal role in regulating reproductive function, thermoregulation, food intake, sexual function, and blood pressure regulation from within the hypothalamus. Research in both animal and human models has demonstrated the critical role of KNDy neurons in these processes. Consequently, the hypothalamus, particularly KNDy neurons, has emerged as a promising therapeutic target for managing VMS.

Although HMT provides effective relief for VMS, its use remains limited due to concerns following the publication of the Women’s Health Initiative (WHI) study results, as well as persistent safety concerns and low awareness regarding HMT [46]. Two newly developed neurokinin (NK) antagonists—fezolinetant (an NK-3 receptor antagonist) and elinzanetant (a dual NK-1/NK-3 receptor antagonist)—offer important non-hormonal alternatives for menopausal symptom management.

A particularly challenging clinical scenario is the treatment of severe VMS in women with a history of breast cancer. Elinzanetant provides new possibilities for these patients by offering a safe, non-hormonal option. Moreover, its potential applications extend to men undergoing androgen deprivation therapy for prostate cancer, where VMS management is equally relevant.

Importantly, fezolinetant and elinzanetant do not act on estrogenic pathways, thereby avoiding risks associated with endometrial hyperplasia, endometrial cancer, and breast tissue malignancy. This favorable safety profile makes NKB antagonists particularly appealing for patients hesitant to use HMT for personal or medical reasons [56]. However, since NKB antagonists do not address urogenital health or bone density loss, combining elinzanetant with low dose HMT in specific clinical scenarios may offer additional benefits.

The development of Neurokinin B antagonist, including NK-3 and dual NK-1/NK-3 receptor antagonists, represents a significant advancement in pharmacological research within gynecological endocrinology. This innovative approach may also have broader implications for other gynecological endocrine disorders. Future investigations could explore the role treatments targeting KNDy neurons—whether through agonist or antagonist pathways—for conditions, such as functional hypothalamic amenorrhea (FHA), polycystic ovary syndrome (PCOS), and endometriosis [46].

Ongoing research into non-hormonal therapies, innovative treatment strategies, and methods to delay the onset of menopause offers a promising outlook for women managing menopausal symptoms. These advancements are poised to significantly improve quality of life for women worldwide and expand their range of available treatment options.

The years ahead hold great promise for further breakthroughs in this field, building on the remarkable progress achieved with NKB antagonists in the treatment of menopausal VMS.

## 8. Conclusions

Neurokinin B receptor antagonists represent a novel class of non-hormonal therapies for VMS. Recent studies show that these NKB receptor antagonists not only alleviate VMS but also improve sleep disturbances and overall quality of life. Beyond menopause, NKB antagonist hold potential as therapeutic options for other gynecological endocrine disorders, including PCOS, uterine fibroids, and endometriosis. Further research in this area may reveal additional benefits and expand the clinical application of these treatments. Future advancements in neurokinin-based therapies will likely provide new, effective, and safer alternatives for managing menopause and related conditions, offering significant improvements in women’s healthcare.

## References

[1] Nagae M., Uenoyama Y., Okamoto S., Tsuchida H., Ikegami K., Goto T., Majarune S., Nakamura S., Sanbo M., Hirabayashi M. (2021). Direct evidence that KNDy neurons maintain gonadotropin pulses and folliculogenesis as the GnRH pulse generator. Proc. Natl. Acad. Sci. USA.

[2] Uenoyama Y., Nagae M., Tsuchida H., Inoue N., Tsukamura H. (2021). Role of KNDy Neurons Expressing Kisspeptin, Neurokinin B, and Dynorphin A as a GnRH Pulse Generator Controlling Mammalian Reproduction. Front. Endocrinol..

[3] Oride A., Kanasaki H. (2024). The role of KNDy neurons in human reproductive health. Endocr. J..

[4] Tsukamura H., Ozawa H., Lehman M.N. (2024). Kisspeptin and mammalian reproduction. Peptides.

[5] Harihar S. (2024). KISS1 and Kisspeptins Detection in Cell Lines. Methods Mol. Biol..

[6] Onsoi W., Numsriskulrat N., Aroonparkmongkol S., Supornsilchai V., Srilanchakon K. (2024). Kisspeptin and DLK1 levels for monitoring treatment of girls with central precocious puberty. Clin. Exp. Pediatr..

[7] Zhou W., Li S., Liu Y., Qi X., Chen H., Cheng C.H., Liu X., Zhang Y., Lin H. (2012). The evolution of tachykinin/tachykinin receptor (TAC/TACR) in vertebrates and molecular identification of the TAC3/TACR3 system in zebrafish (*Danio rerio*). Mol. Cell. Endocrinol..

[8] Wojtas M.N., Diaz-González M., Stavtseva N., Shoam Y., Verma P., Buberman A., Izhak I., Geva A., Basch R., Ouro A. (2024). Interplay between hippocampal TACR3 and systemic testosterone in regulating anxiety-associated synaptic plasticity. Mol. Psychiatry.

[9] Bangalore Krishna K., Witchel S.F. (2024). Normal Puberty. Endocrinol. Metab. Clin. N. Am..

[10] Upton B.A., D’Souza S.P., Lang R.A. (2021). QPLOT Neurons—Converging on a Thermoregulatory Preoptic Neuronal Population. Front. Neurosci..

[11] Moore A.M., Novak A.G., Lehman M.N. (2023). KNDy Neurons of the Hypothalamus and Their Role in GnRH Pulse Generation: An Update. Endocrinology.

[12] Ivanova D., O’Byrne K.T. (2024). New methods to investigate the GnRH pulse generator. J. Mol. Endocrinol..

[13] Moore A.M., Coolen L.M., Lehman M.N. (2022). In vivo imaging of the GnRH pulse generator reveals a temporal order of neuronal activation and synchronization during each pulse. Proc. Natl. Acad. Sci. USA.

[14] Voliotis M., Li X.F., De Burgh R.A., Lass G., Ivanova D., McIntyre C., O’Byrne K., Tsaneva-Atanasova K. (2021). Modulation of pulsatile GnRH dynamics across the ovarian cycle via changes in the network excitability and basal activity of the arcuate kisspeptin network. Elife.

[15] Mittelman-Smith M.A., Krajewski-Hall S.J., McMullen N.T., Rance N.E. (2015). Neurokinin 3 Receptor-Expressing Neurons in the Median Preoptic Nucleus Modulate Heat-Dissipation Effectors in the Female Rat. Endocrinology.

[16] Szeliga A., Podfigurna A., Bala G., Meczekalski B. (2023). Decreased neurokinin B as a risk factor of functional hypothalamic amenorrhea. Gynecol. Endocrinol..

[17] Mittelman-Smith M.A., Williams H., Krajewski-Hall S.J., McMullen N.T., Rance N.E. (2012). Role for kisspeptin/neurokinin B/dynorphin (KNDy) neurons in cutaneous vasodilatation and the estrogen modulation of body temperature. Proc. Natl. Acad. Sci. USA.

[18] Rometo A.M., Krajewski S.J., Lou Voytko M., Rance N.E. (2007). Hypertrophy and Increased Kisspeptin Gene Expression in the Hypothalamic Infundibular Nucleus of Postmenopausal Women and Ovariectomized Monkeys. J. Clin. Endocrinol. Metab..

[19] Patel B., Dhillo W.S. (2022). Menopause review: Emerging treatments for menopausal symptoms. Best Pract. Res. Clin. Obstet. Gynaecol..

[20] Dacks P.A., Krajewski S.J., Rance N.E. (2011). Activation of Neurokinin 3 Receptors in the Median Preoptic Nucleus Decreases Core Temperature in the Rat. Endocrinology.

[21] Ruth K.S., Beaumont R.N., Locke J.M., Tyrrell J., Crandall C.J., Hawkes G., Frayling T.M., Prague J.K., Patel K.A., Wood A.R. (2023). Insights into the genetics of menopausal vasomotor symptoms: Genome-wide analyses of routinely-collected primary care health records. BMC Med. Genom..

[22] Crandall C.J., Manson J.E., Hohensee C., Horvath S.P., Wactawski-Wende J., LeBlanc E.S., Vitolins M.Z.D., Nassir R., Sinsheimer J.S. (2017). Association of genetic variation in the tachykinin receptor 3 locus with hot flashes and night sweats in the Women’s Health Initiative Study. Menopause.

[23] Sun W., Yang F., Zhang H., Yuan Q., Ling S., Wang Y., Lv P., Li Z., Luo Y., Liu D. (2023). Structural insights into neurokinin 3 receptor activation by endogenous and analogue peptide agonists. Cell Discov..

[24] Elder S., Santoro N. (2023). NK3R antagonists: A novel approach for menopause symptoms. Nat. Rev. Endocrinol..

[25] Szeliga A., Czyzyk A., Podfigurna A., Genazzani A.R., Genazzani A.D., Meczekalski B. (2018). The role of kisspeptin/neurokinin B/dynorphin neurons in pathomechanism of vasomotor symptoms in postmenopausal women: From physiology to potential therapeutic applications. Gynecol. Endocrinol..

[26] Johnson K.A., Martin N., Nappi R.E., Neal-Perry G., Shapiro M., Stute P., Thurston R.C., Wolfman W., English M., Franklin C. (2023). Efficacy and Safety of Fezolinetant in Moderate to Severe Vasomotor Symptoms Associated with Menopause: A Phase 3 RCT. J. Clin. Endocrinol. Metab..

[27] Depypere H., Lademacher C., Siddiqui E., Fraser G.L. (2021). Fezolinetant in the treatment of vasomotor symptoms associated with menopause. Expert. Opin. Investig. Drugs.

[28] Tahara A., Takamatsu H., Ohtake A., Tanaka-Amino K., Kaku S. (2021). Effects of neurokinin 3 receptor antagonist fezolinetant on hot flash-like symptoms in ovariectomized rats. Eur. J. Pharmacol..

[29] Fraser G.L., Hoveyda H.R., Clarke I.J., Ramaswamy S., Plant T.M., Rose C., Millar R.P. (2015). The NK3 Receptor Antagonist ESN364 Interrupts Pulsatile LH Secretion and Moderates Levels of Ovarian Hormones Throughout the Menstrual Cycle. Endocrinology.

[30] MacLeay J.M., Lehmer E., Enns R.M., Mallinckrodt C., Bryant H.U., Turner A.S. (2003). Central and peripheral temperature changes in sheep following ovariectomy. Maturitas.

[31] Neal-Perry G., Cano A., Lederman S., Nappi R.E., Santoro N., Wolfman W., English M., Franklin C.B., Valluri U., Ottery F.D. (2023). Safety of Fezolinetant for Vasomotor Symptoms Associated with Menopause: A Randomized Controlled Trial. Obstet. Gynecol..

[32] (2023). FDA Approves Novel Drug to Treat Moderate to Severe Hot Flashes Caused by Menopause.

[33] Szeliga A., Podfigurna A., Bala G., Meczekalski B. (2020). Kisspeptin and neurokinin B analogs use in gynecological endocrinology: Where do we stand?. J. Endocrinol. Invest..

[34] Skorupskaite K., George J.T., Anderson R.A. (2014). The kisspeptin-GnRH pathway in human reproductive health and disease. Hum. Reprod. Update.

[35] Nilsson S., Henriksson M., Berin E., Engblom D., Holm A.-C.S., Hammar M. (2022). Resistance training reduced luteinising hormone levels in postmenopausal women in a substudy of a randomised controlled clinical trial: A clue to how resistance training reduced vasomotor symptoms. PLoS ONE.

[36] McCarthy E.A., Dischino D., Maguire C., Leon S., Talbi R., Cheung E., Schteingart C.D., Rivière P.J.M., Reed S.D., Steiner R.A. (2022). Inhibiting Kiss1 Neurons with Kappa Opioid Receptor Agonists to Treat Polycystic Ovary Syndrome and Vasomotor Symptoms. J. Clin. Endocrinol. Metab..

[37] Oakley A.E., Steiner R.A., Chavkin C., Clifton D.K., Ferrara L.K., Reed S.D. (2015). κ Agonists as a novel therapy for menopausal hot flashes. Menopause.

[38] https://www.drugs.com/monograph/fezolinetant.html.

[39] Onge E.S., Phillips B., Miller L. (2023). Fezolinetant: A New Nonhormonal Treatment for Vasomotor Symptoms. J. Pharm. Technol. jPT Off. Publ. Assoc. Pharm. Tech..

[40] Lederman S., Ottery F.D., Cano A., Santoro N., Shapiro M., Stute P., Thurston R.C., English M., Franklin C., Lee M. (2023). Fezolinetant for treatment of moderate-to-severe vasomotor symptoms associated with menopause (SKYLIGHT 1): A phase 3 randomised controlled study. Lancet.

[41] Cano A., Nappi R.E., Santoro N., Stute P., Blogg M., English M.L., Morga A., Scrine L., Siddiqui E., Ottery F.D. (2024). Fezolinetant impact on health-related quality of life for vasomotor symptoms due to the menopause: Pooled data from SKYLIGHT 1 and SKYLIGHT 2 randomised controlled trials. BJOG Int. J. Obstet. Gynaecol..

[42] (2023). Fezolinetant. Am. J. Heal. Pharm..

[43] (2024). FDA Adds Warning About Rare Occurrence of Serious Liver Injury with Use of Veozah (Fezolinetant) for Hot Flashes Due to Menopause.

[44] https://www.businesswire.com/news/home/20241009445867/en/U.S.-Food-and-Drug-Administration-FDA-accepts-New-Drug-Application-for-elinzanetant.

[45] https://www.drugs.com/newdrugs/fda-approves-veozah-fezolinetant-vasomotor-due-menopause-6013.html.

[46] Meczekalski B., Niwczyk O., Bala G., Szeliga A. (2022). Stress, kisspeptin, and functional hypothalamic amenorrhea. Curr. Opin. Pharmacol..

[47] Simon J.A., Anderson R.A., Ballantyne E., Bolognese J.M., Caetano C., Joffe H., Kerr M., Panay N.F., Seitz C., Seymore S.B. (2023). Efficacy and safety of elinzanetant, a selective neurokinin-1,3 receptor antagonist for vasomotor symptoms: A dose-finding clinical trial (SWITCH-1). Menopause.

[48] Pinkerton J.V., Simon J.A., Joffe H., Maki P.M., Nappi R.E., Panay N., Soares C.N., Thurston R.C., Caetano C., Haberland C. (2024). Elinzanetant for the Treatment of Vasomotor Symptoms Associated with Menopause: OASIS 1 and 2 Randomized Clinical Trials. JAMA.

[49] Pinkerton J.V., Simon J., Panay N., Seitz C., Parke S., Caetano C., Mellinger U., Mashhadi N.H., Haberland C.M., Atanackovic G. (2024). Design of OASIS 1 and 2: Phase 3 clinical trials assessing the efficacy and safety of elinzanetant for the treatment of vasomotor symptoms associated with menopause. Menopause.

[50] Menegaz de Almeida A., Oliveira P., Lopes L., Leite M., Morbach V., Kelly F.A., Barros Í., de Moraes F.C.A., Prevedello A. (2025). Fezolinetant and Elinzanetant Therapy for Menopausal Women Experiencing Vasomotor Symptoms: A Systematic Review and Meta-analysis. Obstet. Gynecol..

[51] Fraser G.L., Obermayer-Pietsch B., Laven J., Griesinger G., Pintiaux A., Timmerman D., Fauser B.C.J.M., Lademacher C., Combalbert J., Hoveyda H.R. (2021). Randomized Controlled Trial of Neurokinin 3 Receptor Antagonist Fezolinetant for Treatment of Polycystic Ovary Syndrome. J. Clin. Endocrinol. Metab..

[52] Guo F., Fernando T., Zhu X., Shi Y. (2023). The overexpression of neurokinin B-neurokinin 3 receptor system exerts direct effects on the ovary under PCOS-like conditions to interfere with mitochondrial function. Am. J. Reprod. Immunol..

[53] Iyer T.K., Fiffick A.N., Batur P. (2024). Nonhormone therapies for vasomotor symptom management. Clevel. Clin. J. Med..

[54] McNeil M.A., Merriam S.B. (2021). Menopause. Ann. Intern. Med..

[55] Ohtaki T., Shintani Y., Honda S., Matsumoto H., Hori A., Kanehashi K., Terao Y., Kumano S., Takatsu Y., Masuda Y. (2001). Metastasis suppressor gene KiSS-1 encodes peptide ligand of a G-protein-coupled receptor. Nature.

[56] Harper-Harrison G., Carlson K., Shanahan M.M. (2025). Hormone Replacement Therapy. StatPearls.

